# Programa de Redução de Estresse, Meditação e Mindfulness para Pacientes com Insuficiência Cardíaca: Um Pouco de Luz na Escuridão

**DOI:** 10.36660/abc.20230713

**Published:** 2023-11-14

**Authors:** Luís Beck-da-Silva

**Affiliations:** 1 Departamento de Medicina Interna Universidade Federal do Rio Grande do Sul Porto Alegre RS Brasil Departamento de Medicina Interna da Universidade Federal do Rio Grande do Sul (UFRGS), Porto Alegre, RS – Brasil; 2 Serviço de Cardiologia Hospital de Clínicas de Porto Alegre Porto Alegre RS Brasil Serviço de Cardiologia do Hospital de Clínicas de Porto Alegre (HCPA), Porto Alegre, RS – Brasil

**Keywords:** Ensaio Clínico Randomizado, Insuficiência Cardíaca, Meditação, Terapia Cognitivo-Comportamental, Atenção Plena

O estudo intitulado “Impacto de um Programa de Redução do Estresse, Meditação e *Mindfulness* em Pacientes com Insuficiência Cardíaca Crônica: Um Ensaio Clínico Randomizado”^1^ levantou um assunto muito importante a ser abordado em pacientes com insuficiência cardíaca e pretendeu fazê-lo através da realização de um ensaio clínico randomizado. O impacto do estresse, da depressão, da falta de propósito de vida e das alterações psicológicas dos pacientes impostas por uma doença crônica e debilitante contribui definitivamente para a piora da qualidade de vida e possivelmente para um pior prognóstico.^2,3^ Avaliar este impacto e pesquisar possíveis terapias é profundamente necessário. Os autores devem ser reconhecidos pelo seu interesse e enorme esforço na condução de um ensaio tão laborioso.

O estudo,^1^ no entanto, apresenta algumas deficiências. Embora os autores afirmem ser um ensaio de insuficiência cardíaca com fração de ejeção reduzida (ICFEr), de fato, o estudo incluiu aproximadamente metade dos pacientes com ICFEr. Apesar da heterogeneidade da amostra, um terço dos pacientes no grupo de intervenção foram perdidos no acompanhamento, enquanto nenhum paciente foi perdido no grupo controle. Portanto, mesmo tendo selecionado mais de duzentos indivíduos, apenas 13 pacientes foram efetivamente submetidos à intervenção. Os autores não nos informam como esses pacientes foram tratados farmacologicamente, mas como o estudo foi realizado há quatro anos, pode-se presumir que o tratamento dos pacientes estava desatualizado.

O estudo^1^ teve como objetivo principal avaliar o impacto de um programa baseado na redução do estresse, meditação e técnicas de *mindfulness* no estresse percebido pelos pacientes. Aparentemente, o estudo foi positivo na medida em que o “estresse percebido”, avaliado pela escala de autorrelato PSS-14, foi significativamente reduzido através de qualquer uma das análises. É preciso estar ciente de que este é um estudo aberto, onde um grupo recebeu oito sessões semanais de duas horas sobre técnicas de redução do estresse, enquanto o grupo controle não recebeu nenhuma intervenção. O objetivo principal é pedir a ambos os grupos que autoavaliem o estresse percebido. O viés do efeito placebo é irremovível a partir deste resultado. Outros questionários utilizados como objetivos secundários no estudo estão sob o mesmo viés.

Houve um aparente aumento na distância percorrida no teste de caminhada de 6 minutos (T6MC), embora os autores tendam a destacar a mudança significativa pré e pós-intervenção no grupo intervenção, mas nenhuma mudança aparente no delta entre os grupos.

Os níveis séricos de cortisol entraram no texto, alegando um resultado estatisticamente significativo. Isso sem entrar, é claro, na relevância clínica de uma variação de 0,4 mcg/dL no nível sérico de cortisol.

Finalmente, as medidas laboratoriais objetivas que poderiam nos dar uma ideia da melhora real na insuficiência cardíaca do paciente, como a proteína C reativa (PCR) e o peptídeo natriurético (NT-ProBNP), permaneceram inalteradas.

O estresse e o sofrimento psicológico dos nossos pacientes com insuficiência cardíaca são de extrema importância na nossa prática clínica e este aspecto não deve ser esquecido. O estudo publicado nesta edição da ABC^1^ traz luz ao assunto e sugere que os pacientes possam se beneficiar de alguma forma com um programa de redução de estresse, meditação e *mindfulness*. Este estudo pode contribuir como fonte de dados para futuros estudos maiores, com algumas análises de dados cegantes e minuciosas, para finalmente concluir o impacto dessas técnicas no prognóstico da insuficiência cardíaca.
