# Supplementary material for: Programa de Redução de Estresse, Meditação e Mindfulness para Pacientes com Insuficiência Cardíaca: Um Pouco de Luz na Escuridão
Source: Arq Bras Cardiol. 2023 Nov 14;120(10):e20230713. [Article in Portuguese] doi: 10.36660/abc.20230713 (PMC10697685; doi:10.36660/abc.20230713)
Supplement: Translation

# Translation: Stress Reduction, Meditation and Mindfulness Program for Heart Failure Patients: Some Light in the Darkness

**Authors:** Luís Beck-da-Silva

**Affiliations:** 1 Brazil Departamento de Medicina Interna da Universidade Federal do Rio Grande do Sul (UFRGS), Porto Alegre, RS – Brazil; 2 Brazil Serviço de Cardiologia do Hospital de Clínicas de Porto Alegre (HCPA), Porto Alegre, RS – Brazil

**Keywords:** Randomized Controlled Trial, Heart Failure, Meditation, Cognitive Behavioral Therapy, Mindfulness

The study entitled “Impact of a Stress Reduction, Meditation, and Mindfulness Program in Patients with Chronic Heart Failure: A Randomized Controlled Trial”^1^ has raised a very important subject to be addressed in patients with heart failure and aimed to do so by conducting a randomized clinical trial. The impact of patients’ stress, depression, lack of life purpose, and psychological changes imposed by a chronic and debilitating disease definitely contributes to worsening quality of life and possibly to worse prognosis.^2,3^ To assess this impact and to research possible therapies is deeply needed. The authors should be recognized for their interest and huge effort in conducting such a laborious trial.

The study,^1^ however, has some deficiencies. Although the authors claim to be a heart failure with reduced ejection fraction (HFrEF) trial, the study included approximately half of the patients with HFrEF. Despite the heterogeneity of the sample, one-third of patients in the intervention group were lost to follow-up, while no patients were lost in the control group. Therefore, even having screened more than two hundred individuals, only 13 patients were actually submitted to intervention. The authors do not inform us of how these patients were pharmacologically treated, but given that the study was conducted four years ago, one can assume patients’ treatment was outdated.

The study^1^ aimed primarily to evaluate a program’s impact based on stress reduction, meditation, and mindfulness techniques on patients’ perceived stress. Apparently, the study was positive in that “perceived stress,” assessed by the PSS-14 self-report scale, has been significantly reduced through any of the analyses. One must be aware that this is an open-label study, where one group received eight weekly two-hour sessions on stress reduction techniques while the control group received no intervention. The primary objective is to ask both groups to self-assess their perceived stress. The bias of the placebo effect is indefeasible from this result. Other questionnaires used as secondary objectives in the trial are under the same bias.

There was an apparent increment in the distance walked in the 6-min walk test (T6MC), although authors tend to highlight the significant change from pre and post-intervention in the intervention group but no apparent change in the delta between groups.

Serum cortisol levels entered the text, claiming a statistically significant result. This is without going into, of course, the clinical relevance of a variation of 0.4 mcg/dL in the serum cortisol level.

Finally, the objective lab measures which could give us an idea of actual improvement in patient’s heart failure, such as C-reactive protein (CRP) and natriuretic peptide (NT-ProBNP), remained unchanged.

Our heart failure patients’ stress and psychological suffering are of utmost importance in our clinical practice, and this aspect must not be forgotten. The study published in this issue of ABC^1^ brings light to the subject and suggests that patients may somewhat benefit from a stress reduction, meditation, and mindfulness program. This study can contribute as a source of data for future larger trials, with some blinding and thorough data analysis, to finally conclude the impact of these techniques on the prognosis of heart failure.
